# Soil bacteria as sources of virulence signal providers promoting plant infection by *Phytophthora* pathogens

**DOI:** 10.1038/srep33239

**Published:** 2016-09-12

**Authors:** Ping Kong, Chuanxue Hong

**Affiliations:** 1 Hampton Roads Agricultural Research and Extension Center, Virginia Tech, 1444 Diamond Springs Road, Virginia Beach, VA 23455, USA

## Abstract

*Phytophthora* species are known as “plant destroyers” capable of initiating single zoospore infection in the presence of a quorum of chemical signals from the same or closely related species of oomycetes. Since the natural oomycete population is too low to reach a quorum necessary to initiate a disease epidemic, creation of the quorum is reliant on alternate sources. Here, we show that a soil bacterial isolate, *Bacillus megaterium* Sb5, promotes plant infection by *Phytophthora* species. In the presence of Sb5 exudates, colonization of rhododendron leaf discs by 12 *Phytophthora* species/isolates was significantly enhanced, single zoospores of *P. nicotianae* infected annual vinca and *P. sojae* race 25 successfully attacked a non-host plant, *Nicotiana benthamiana* as well as resistant soybean cultivars with *RPS1a* or *RPS3a*. Sb5 exudates, most notably the fractions larger than 3 kDa, promoted plant infection by improving zoospore swimming, germination and plant attachment. Sb5 exudates also stimulated infection hypha growth and upregulated effector gene expression. These results suggest that environmental bacteria are important sources of virulence signal providers that promote plant infection by *Phytophthora* species, advancing our understanding of biotic factors in the environmental component of the Phytophthora disease triangle and of communal infection of plant pathogens.

*Phytophthora,* a genus in Oomycetes of the Kingdom Stramenopila, morphologically resemble fungi but are phylogenetically close relatives of brown algae. This genus includes over 150 species and has diverse life strategies for survival, colonization and pathogenesis^1^,^2^,^3^,^4^,^5^,^6^,^7^. The genus name is of Greek origin meaning “plant destroyer” as many *Phytophthora* species are destructive pathogens of agriculturally and economically important plants, causing significant crop loss and damage to natural ecosystems^8^,^9^,^10^.

The process of plant infection by these pathogens, however, is not well understood. Motile asexual zoospores are the principal infective weapons of *Phytophthora* species^4^. In general, a quorum of zoospores is required to produce adequate chemical signals to trigger infection^11^. This quorum as determined in the laboratory ranges from 100 to 10^5^zoospores/ml or higher depending on the species^12^,^13^,^14^,^15^. This range is well above the natural populations that have caused disease problems in the field but are difficult to detect with sensitive molecular diagnostic methods^16^,^17^. These pathogens must have developed mechanisms to allow them to infect plants at such low inoculum concentrations in nature. We recently demonstrated that a single *Phytophthora* zoospore successfully attacked a plant in the presence of a quorum of chemical signals from the same or other closely related zoosporic oomycetes including *Pythium*^11^,^18^. However, the total population of zoosporic oomycetes in nature remains relatively low^17^,^19^,^20^, and it alone is unlikely to reach the quorum required to trigger plant infection by *Phytophthora* species.

We hypothesized that there are alternative resources for quorum and virulence acquisition in plant infection by *Phytophthora* species with bacteria topping the candidate list. First, bacteria are widespread, they grow exponentially, and their population sizes are unbeatable. Bacteria in soil are estimated to be 2,000 to 8.3 million per gram^21^,^22^. Second, bacteria coexist with *Phytophthora* species in a wide variety of environments including soil, water, and plants, and they also are frequently isolated from “purified” *Phytophthora* culture storage (Kong *et al*. unpublished data). Third, *Phytophthora* species produces the bacterial universal quorum sensing signal AI-2 that may be recruiters of bacteria for Phytophthora pathogens^23^,^24^. Fourth, bacteria have a variety of secretion systems that release metabolites into the environment^25^,^26^. Some of metabolites play important roles in the life cycle of *Phytophthora* species. They enhance the growth and reproduction of *Phytophthora* species^27^,^28^,^29^,^30^. They also may act as plant hormone mimics stimulating the opening of plant stomata to allow pathogens to enter the apoplast and impair defense gene expression^31^,^32^,^33^,^34^. They may also act as transformers of plant products promoting infections by these pathogens. For example, bacteria can convert isoflavones produced by soybean plants, into a substance that is 100 times more attractive to zoospores of *P. sojae*^35^, consequently facilitating zoospore aggregation on or around plant surface, a prerequisite of infection process. Similarly, plant colonization by *Phytophthora* species is associated with abundant bacterial populations in the rhizosphere^36^. However, such beneficial impacts of environmental microorganisms on plant pathogens are largely overlooked in current research^24^. Instead, negative or detrimental impacts have been a focus aiming to identify a biocontrol agent for the diseases although effective products are rare^37^,^38^,^39^.

In contrast, the importance of a beneficial relationship between pathogens and the surrounding microorganisms has been better recognized in medical science^40^,^41^. For example, infection by *Candida albicans* in humans has been linked to co-colonized bacterial biofilms^42^. Infection of immunocompromised patients by *Cryptococcus neoformans* is attributed to virulence precursors provided by *Klebsiella aerogenes* bacterial microflora^43^. These studies have provided unparalleled insights into pathogen ecology, pathogenicity, and disease epidemiology, facilitating our understanding of how plant infections occur under natural conditions and ways to counteract.

Here, we explore the beneficial effects of soil bacterial exudates on *Phytophthora* species during plant infection. We focus on a soil isolate of *Bacillus megaterium*, Sb5, and demonstrate that environmental bacteria are sources of signals for *Phytophthora* virulence as well as fitness, and communal infection is a life strategy of these pathogens.

## Results

### Soil bacteria enhance the colonization of rhododendron leaf discs by *Phytophthora* species

**Figure 1:**
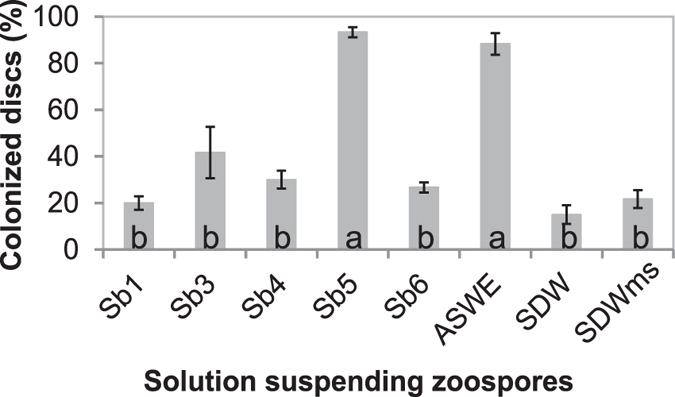
Soil bacterial products enhance colonization of rhododendron leaf discs by *Phytophthora nicotianae*. Leaf discs were submerged in a suspension at 10 zoospores/ml in an autoclaved filtrate of bacterial cell suspension of individual isolates (Sb1, Sb3, Sb4, Sb5 or Sb6) prepared from potato dextrose agar (PDA), autoclaved soil water extract (ASWE), sterile distilled water (SDW) or rinsate of PDA plate surface with SDW (SDWms). The discs were examined for colonization 48 h after being plated on PARP-V8 agar. The graph depicts colonization rates (%) of the treatments. Each column is a mean of three repeating experiments. Bars depict standard error. Columns with the same letter are not different according to LSD test at *P* = 0.05.

In an initial assay with *P. nicotianae* isolate 1B11, soil water extract (SWE) and its five major bacterial component species (Sb1, 3 to 6) were compared with sterile distilled water (SDW) and surface rinsate of potato dextrose (PDA) agar medium used to grow bacteria (SDWms) for their impact on pathogen colonization. This was evaluated by submerging leaf discs of Rhododendron, a universal suscept, overnight in zoospore suspensions prepared with autoclaved bacterial cell suspensions of individual isolates in PDA plates, autoclaved SWE (ASWE), SDW or SDWms. Sb5 and ASWE significantly enhanced colonization of leaf discs by *P. nicotianae* when compared to SDW (Fig. 1). In contract, the other 4 bacterial isolates (Sb 1, 3, 4 and 6), and SDWms had no impact on the Phytophthora colonization.

To confirm the activity of Sb5, additional tests were conducted with 11 other Phytophthora species/isolates. Sb5 significantly promoted disc colonization by all the species and isolates assessed (Table 1). ASWE was also included for the purpose of comparison. As expected, it also promoted disc colonization by all the species and isolates.

### Sb5 exudates promote plant infection by *Phytophthora* species

**Figure 2:**
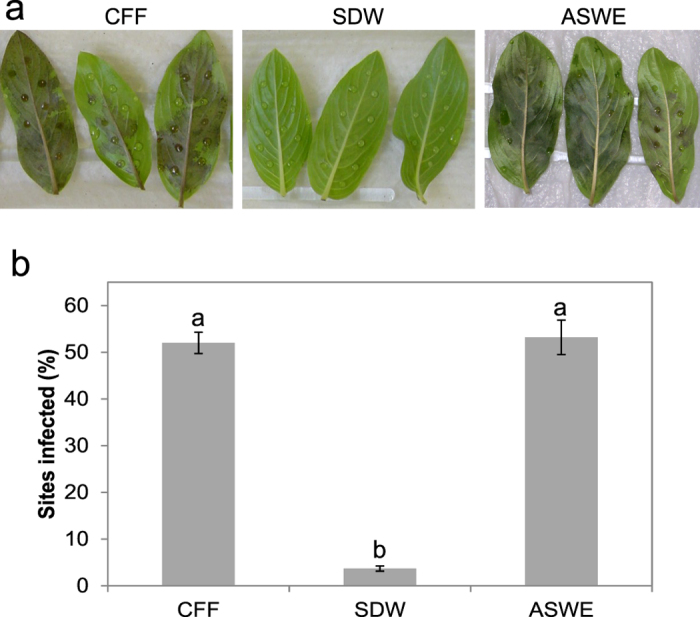
Sb5 cell-free filtrate (CFF) promotes infection of annual vinca (*Catharanthus roseus*) leaves by single zoospores of *Phytophthora nicotianae*. Individual leaves were inoculated at ten different sites each with a 10-μl drop of zoospore suspension (100/ml) prepared with CFF, sterile distilled water (SDW) or autoclaved soil water extract (ASWE). (**a)** Symptoms and (**b**) Infection rates at 72 h after inoculation. Each column is a mean of sites infected from three repeating experiments. Bars depict standard error. Columns with the same letter are not different according to LSD test at *P* = 0.05.

Sb5 was further assessed with the cell-free filtrate (CFF) for its ability to promote infection of whole leaves and intact plants by *Phytophthora* species using three phytopathosystems: annual vinca - *P. nicotianae*, soybean - *P. sojae,* and tobacco - *P. sojae*. In the annual vinca - *P. nicotianae* system, a single zoospore infection assay^11^ was performed with detached leaves to determine whether Sb5 extracellular products may be used as quorum sensing signals by the pathogen to initiate infection. *P. nicotianae* caused extensive necrotic lesions on leaves 72 h after inoculation in the CFF treatment but did not in the negative control with SDW (Fig. 2a). Similarly, the percent sites blighted was significantly greater in the CFF treatment than negative controls (Fig. 2b). Like CFF, ASWE had the same level of infection-promoting effect (Fig. 2). Thus, the bacterial extracellular products of Sb5 did promote the quorum sensing-mediated infection by *P. nicotianae*.

**Figure 3:**
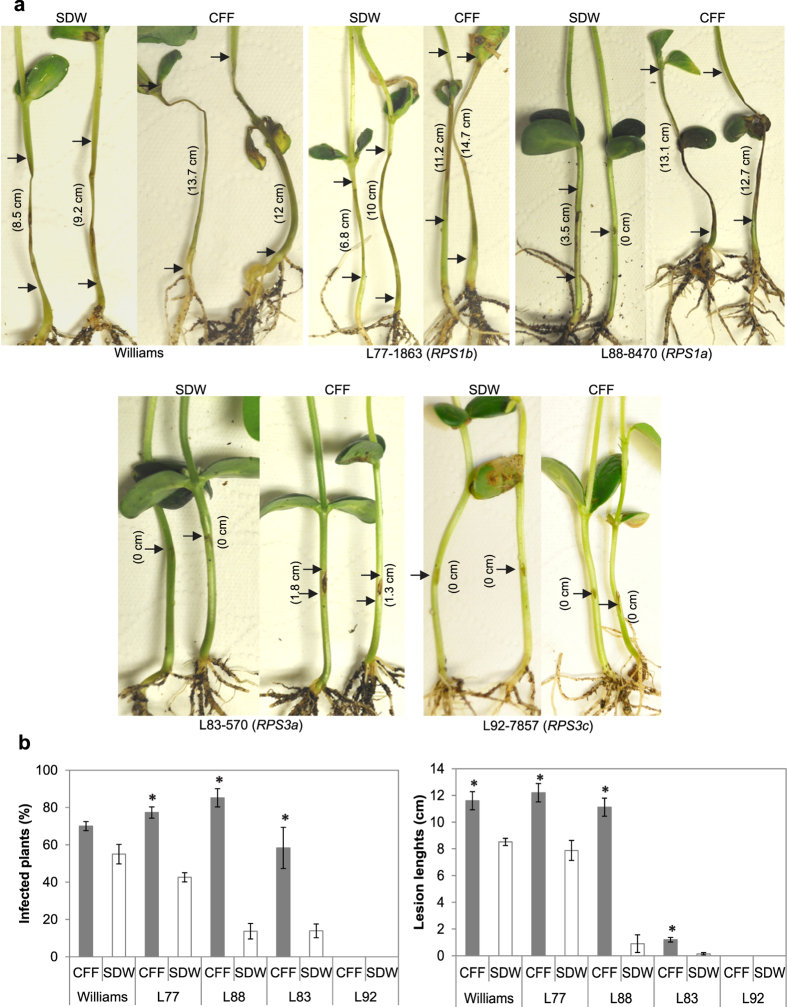
Sb5 cell-free filtrate (CFF) affects the responses of soybean differentials to *Phytophthora sojae* race 25. (**a**) Hypersensitive response of hypocotyls 4 days after being injected with the pathogen mycelia suspended in CFF or sterile distilled water (SDW). Length of lesion at the inoculated site is marked between arrows. CFF treatment resulted in expanded lesions on two susceptible varieties (Williams and L77-1863) and one resistant variety (L88-8470) and necrotic lesions on the resistant variety L83-570 but not L92-7857. (**b**) Infection rate and lesion size are means of three repeating experiments. Bars in columns depict standard errors. *depicts significant difference between the CFF treatment and the SDW control according to t-test assuming unequal variances (*P* = 0.05).

In the soybean - *P. sojae* system, responses of five differential soybean plants to its counterpart *P. sojae* race 25 were examined. Susceptible Williams and L77-1863 plants developed significantly larger lesions and had greater infection rates in the CFF treatment than the SDW control (Fig. 3). Similar infection-promoting effect was observed in two of the three resistant differential plants. L88-8470 plants had significantly larger lesions and higher infection rates in the CFF treatment than the controls, so did L83-570 (Fig. 3). The only exception was L92-7857 plants carrying *RPS3c* resistance gene; no infection developed in either treatment (Fig. 3). These results indicate that CFF altered the interaction between race 25 of *P. sojae* and the differential plants except for L92-7857 with *RPS3c*.

**Figure 4:**
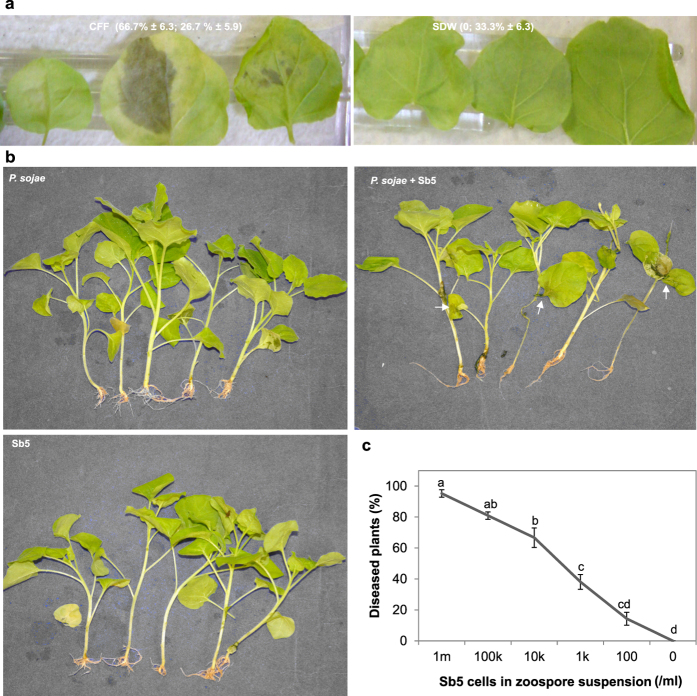
Sb5 cell-free filtrate (CFF) promotes infection of a nonhost plant, *Nicotiana benthamiana* by *Phytophthora sojae*. (**a)** Symptoms on detached leaves 72 h after inoculation on four sites of a leaf with each site having a 20-μl drop containing 250 zoospores in CFF or SDW. (**b)** Disease symptoms (arrows) developed on seedlings after their roots were submerged in a mixture suspension of *P. sojae* at 3,200 zoospores/ml and Sb5 at 10^6^ cells/ml for 6 days but not on any other seedlings whose roots were submerged in suspension of Sb5 or *P. sojae* alone at the same concentration. (**c**) Percent seedlings diseased after their roots were submerged in mixture suspensions of zoospores (Zsp) at 3,200/ml and different concentrations of Sb5 from 10^6^ to zero cell/ml. Each point is a mean of three replicates. Means with the same letter are not different according to LSD test at *P* = 0.05.

In the *Nicotiana benthamiana* - *P. sojae* system, CFF was evaluated for its impact on the interaction between a nonhost plant and a *Phytophthora* species. Nearly 70% of detached leaves were infected when inoculated with zoospores suspended in CFF. Blight symptoms developed 3 days after inoculation (Fig. 4a left). In contrast, the control leaves developed no symptoms or slight yellowing (Fig. 4a right). *P. sojae* was isolated from surface-sterilized leaf tissue with the blight symptom as confirmed using ITS-SSCP analysis of resultant cultures (Fig. S1) but did not from that with the yellowing symptom. These results indicate that *P. sojae* was the causal agent of the symptom and CFF triggered leaf infection on a nonhost plant by *P. sojae*.

CFF also triggered infection of *N. benthamiana* seedlings by *P. sojae*. *N. benthamiana* seedlings did not develop any disease symptoms after the roots were submerged for a week in the suspension of either *P. sojae* at 3,200 zoospores/ml or Sb5 at 10^6^ cells/ml alone. In contrast, those with roots submerged in a mixture suspension of *P. sojae* zoospores and Sb5 cells at the same concentrations developed severe damping-off and blight symptoms. The number of diseased plants decreased with decreasing bacterial concentration in the mixture; Sb5 cells at ≤1,000/ml did not result in a significant difference from the control (Fig. 4c). As from infected leaves, *P. sojae*, but not the bacterium, was isolated from surface-sterilized roots of diseased plants. This again confirmed that *P. sojae* but not Sb5 was the causal agent of the disease, and Sb5 was a stimulator of *N. benthamiana* infection by *P. sojae*.

### Sb5 exudates stimulate zoospore homing and morphogenesis

To understand how CFF facilitated plant infection by *Phytophthora*, zoospore behaviors associated with infection process were examined. Zoospores of all the *Phytophthora* species tested exhibited prolonged swimming in CFF, 14–20 h longer than those in SDW. In the case of *P. nicotianae*, 90% zoospores maintained swimming for at least 2 h in CFF. Among these, about 10% kept swimming for longer than 24 h. In contrast, the majority of zoospores in SDW encysted in less than 2 h, and only few of them made overnight. In the case of *P. sojae*, promotion of zoospore swimming by CFF was not as evident. Many zoospores encysted before the treatment, indicating that zoospore encystment of treatments was not resulted from osmotic shock.

**Figure 5:**
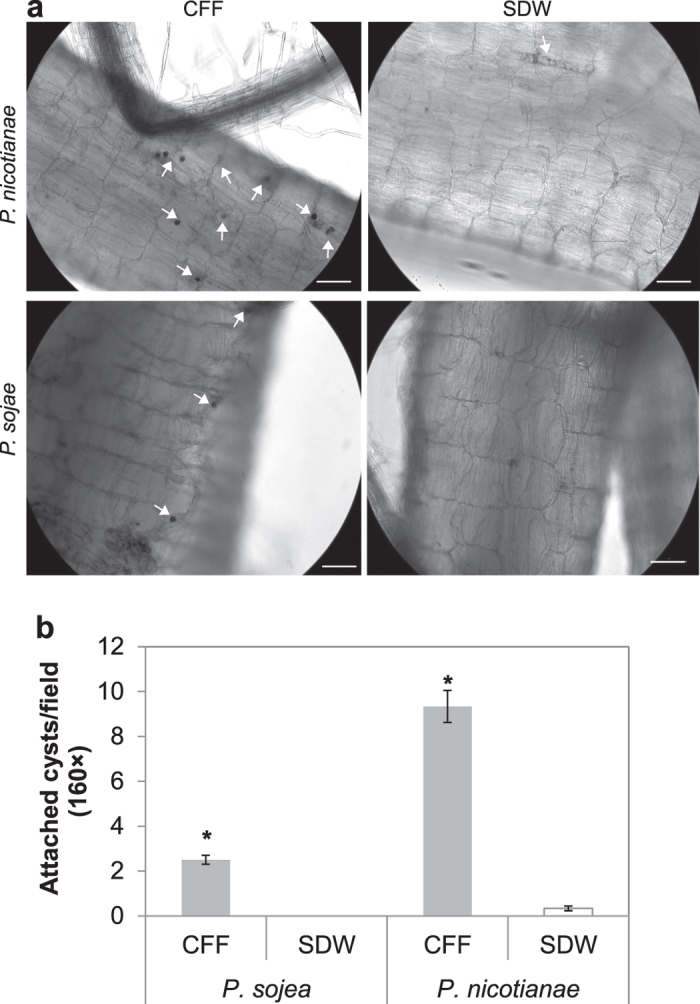
Effect of Sb5 cell-free filtrate (CFF) on zoospore attachment to *Arabidopsis thaliana* (Col-0) roots. (**a**) Photomicrographs taken after roots were incubated 40 min in suspension of *P. nicotianae* or *P. sojae* at 1,000 zoospores/ml prepared with CFF or SDW. Attached cysts were indicated with arrows. Bars = 50 μm. (**b**) Number of attached cysts per microscopic field. Each column is a mean of six fields. *depicts significant difference between the CFF treatment and SDW controls according to t-test assuming equal variances (*P* = 0.05).

More zoospores attached to the root surface of *Arabidopsis* in the presence of CFF (Fig. 5). Approximately nine cysts of *P. nicotianae* and three cysts of *P. sojae* were observed per microscopic field on root surface after 40-min incubation in CFF. Significant fewer cysts attached in the control (Fig. 5b). Number of attached cysts was also different between species. Fewer *P. sojae* zoospores attached to the roots compared to *P. nicotianae* within the same treatment, which may be associated with early encystment of *P. sojae*.

**Figure 6:**
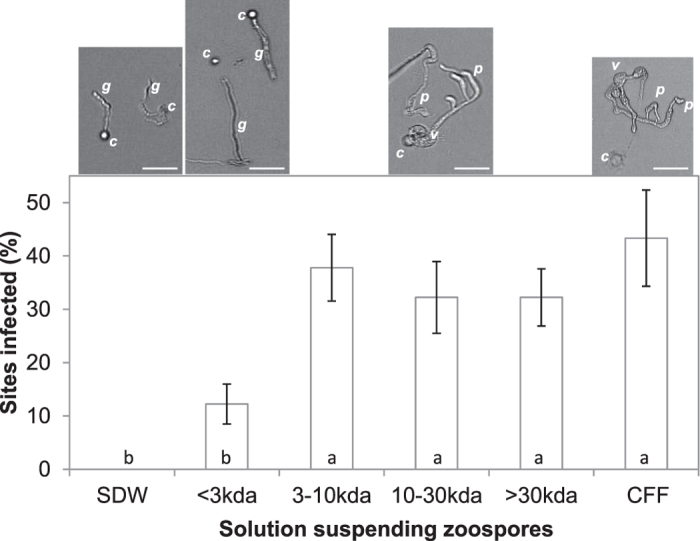
Effect of Sb5 cell-free filtrate (CFF) fractions on zoospore morphogenesis and infection of annual vinca (*Catharanthus roseus*) by *Phytophthora nicotianae*. **Top**: Photomicrographs of cyst morphogenesis taken after 16-h incubation of 500 zoospores in 100 μl SDW, CFF or one of the four CFF fractions on a depression slide. Finger-like projections and vesicles formed in CFF and its fractions with molecular weight >3 kDa. *c, g, p* and *v* indicate cyst, germling, finger-like projections and vesicles, respectively. Bar = 50 μm. **Bottom**: Percent leaf sites infected following inoculation with ten 10 μl-drops of SDW, CFF or one of its fractions with each containing approximately 1 zoospore and 72-h incubation at 23 °C. Each column represents a mean of three repeating experiments. Bars depict standard errors. Columns with the same letter are not statistically different according to LSD at *P* = 0.05.

Cyst germination was also improved by CFF. Germination of *P. nicotianae* and *P. sojae* after 24 h in SDW was 33% and zero, while in CFF it was 55% and 75%, respectively. The process after germination also benefited from CFF. Cyst morphogenesis underwent upon further incubation in CFF, developing robust finger-like projections, but not in SDW where short and thin projections formed (Fig. S2). Formation of these structures was associated with successful plant infection by zoospores. In the annual vinca - *P. nicotianae* system, plant infection was significantly enhanced when zoospore inoculum was prepared with CFF but not with SDW (Fig. 6).

### Sb5 CFF regulates expression of effector genes of *P. sojae*

**Figure 7:**
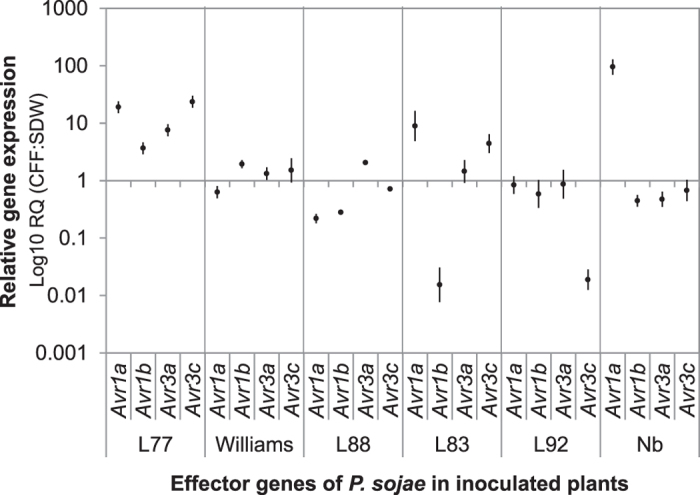
Effector gene expression in plants after inoculation with and without presence of Sb5 cell-free filtrate (CFF). Real-time PCRs were conducted with RNA from hypocotyls of *Phytophthora sojae* race 25 soybean differentials (L77-1863, L83-570, L88-8470, L92-7857 and Williams) inoculated with mycelia of *P. sojae* mixed with CFF or sterile distilled water (SDW), and from *Nicotiana benthaminana* roots (Nb) that were submerged in a mixture suspension of *P. sojae* at 3,200 zoospores/ml and Sb5 at 10^6^ cells/ml or SDW. Average Ct (threshold cycles) of PCR runs with *P. sojae* effector genes *Avr1a*, *Avr3a*, *Avr1b1* or *Avr3c* in both CFF treatment and control was normalized with mean value of the average Ct with three reference genes, *actin A*, *β-tubulin* and *ubiquitin* in the same sample (dCt = Ct_*effector*_ − Ct_*reference*_). Transcription levels of effector genes in the CFF treatment were measured with 2^−ddCt^ after the calibration of the dCt with that of the control (ddCt = dCt_Sb5_ − dCt_control_). Each point is a mean of three PCR runs. Vertical lines depict the range of transcript level of individual genes by plant and cultivar.

To further understand how CFF may promote the interactions of *P. sojae* race 25 with its differential host plants and a nonhost plant, *N. benthaminana*, RNA from plants 48 h after inoculation in the CFF treatment and controls was examined for expression of *P. sojae* RXLR effector genes, *Avr1a*, *Avr1b*, *Avr3a* and *Avr3c.* All plants of the CFF treatment except for those of L92-7857 had transcript level elevation of one or more effector genes when compared to the controls (Fig. 7). In the L77-1863, all four effector genes were upregulated, with *Avr1a* and *Avr3c* elevated about 20 fold over the control. In the Williams, except for *Avr1a*, all other three genes were upregulated at low levels. Similarly, in the L83-570, except for *Avr1b*, other three genes were upregulated with *Avr1a* and *Avr3c* that elevated more than 5 fold. In the L88-8470 and nonhost tobacco plants, only one effector gene each was upregulated. Moreover, the elevated gene in L88-8470 was not *Avr1a*, an effector gene of *P. sojae* corresponding to *RPS1a*, the R gene of the plant, but was *Avr3a* that elevated two fold over the control, indicating CFF*-*triggered *RPS1a* recognition by *Avr3a*. The elevated gene in the tobacco was *Avr1a* that elevated near 100 fold over the control. This result indicates that upregulation of *Avr1a* allowed the pathogen to recognize R genes of this nonhost which facilitated tobacco plant infection. In contrast, none of effector genes elevated in inoculated L92-7857 that did not produce any symptoms with or without CFF, indicating that CFF did not affect the interactions between L92-7857 with *RPS3c* and race 25. Therefore, CFF-promoted effector gene expression appeared to be dependent on plant resistance genotypes.

### Identification of Sb5 and active molecular size range of CFF

Sb5 was only one tested as Gram positive among five dominant isolates from soil water extracts. This was confirmed with fatty acid methyl ester analysis (FAME) which showed that Sb5 had a similarity index of 89% to Gram positive *Bacillus megaterium* while the other four isolates Sb1, 3, 4 and 6 all were Gram negative bacteria: *Klebsiella pneumoniae*, *Klebsiella ornithinolytica*, *Serratia liquefaciens*, *and Burkholderia ceribiensis*, respectively. Identity of Sb5 was further confirmed by sequencing16S rDNA of the bacterium. The obtained partial sequence (accession number: FJ858254) was 99% identical to that of a *Bacillus megaterium* strain (accession number: NC014103.1) at a query coverage of 100% and E value of 0 in GenBank. Consequently, Sb5 was identified as *Bacillus megaterium*.

The size range of functional components of Sb5 CFF was determined by fractionation through Centriprep columns at molecular weight cutoff of 3, 10 or 30 kDa. Four fractions were obtained and examined for activities in promoting *P. nicotianae* zoospore behaviors *in vitro* and plant infection in the annual vinca - *P. nicotianae* system. All of the fractions prolonged zoospore swimming as CFF. They also promoted zoospore germination. However, only fractions of >3 kDa stimulated zoospore morphogenesis or formation of finger-like projections and vesicles (Fig. 6 top). Likewise, these fractions with larger molecular weight enhanced single zoospore infection of annual vinca, resulting in significantly higher infection rates compared with the smaller fraction and control (Fig. 6 bottom). These results indicate that substances in small and large fractions contributed differently in the different steps of the plant infection process.

## Discussion

This study with *Bacillus megaterium,* a widespread soil bacterium^44^,^45^,^46^, demonstrates that environmental bacteria are sources of virulence signal providers for *Phytophthora* pathogens in plant infection. It advances our understanding of the roles of biotic environmental factor in the interaction between plant and pathogen, providing important new insights into disease development, a collective result of the interaction of susceptible host, virulent pathogen, and conducive environment^47^. We demonstrate that *Phytophthora* species can exploit environmental bacteria to improve their pathogenesis and fitness like fungal pathogens^24^,^40^. In the presence of bacterial metabolites, they can not only increase growth and proliferation^28^,^29^,^30^,^46^, but also improve zoospore homing and colonization as indicated in this study to enhance their likelihood of successful plant infection. They can also initiate or improve an infection in plants with bacteria. *P. nicotianae* initiated its infection at single zoospore level, and *P. sojae* broke up Nonhost defense barriers to infect tobacco plants as shown in this study. Strikingly, *Phytophthora* species have the ability to recruit these ubiquitous microorganisms for their benefits because they can produce the bacterial universal quorum sensing signal^23^. Given such beneficial relationships with bacteria or ability for communal infection, it becomes clearer why *Phytophthora* species remain the most destructive plant pathogens with relatively low populations in nature. More research on relationships between *Phytophthora* and the beneficial phytobiome as well as the chemistry of functional exudates is warranted to facilitate innovation of plant disease control technologies.

This also advanced our understanding of the natural functions of *B. megaterium*. Densities of *B. megaterium* in soil of cropping systems range between 2–6 log cfu/g^44^ which is within the range that promoted plant infection by *Phytophthora* pathogens as demonstrated in this study. On the other hand, *B. megaterium* have been shown to suppress plant infection by *P. capsici*^39^. This inconsistency may be attributed to different modes of action of the bacterium created with different procedures. In the biocontrol experiment bacterial, rinsed bacterial cells at greater than 10^8^/ml were used prior to plant to be challenged with *P. capsici*^39^. This procedure may lead to occupation or blockage of infection sites prior to arrival of the challenging pathogen due to outcompeting of a high density of bacterial cells or other indirect antagonisms^44^,^48^,^49^. In contrast, in this study, exudates or metabolites of the bacterium at much lower concentrations (≤10^6^ cells/ml) were used simultaneously with test Phytophthora to challenge plants. This procedure allows both microorganisms compete at the same time at population densities close to that in nature where the bacterial metabolites may act as mediators of intercellular signaling of virulence and cell differentiation to these pathogens. Metabolites of many bacteria possess a dual nature at different concentrations in the natural ecosystems; they function as antibiotics at high concentration on target microorganisms but as mediators of intercellular signaling of virulence and cell differentiation to these microorganisms at their natural densities^50^. Some bacilli have been shown to produce antibiotics that are antagonistic to *Pythium*^48^, a close relative of *Phytophthora*. It is interesting to see how these antibiotics behavior at subinhibitory concentrations. However, because we did not test a concentration greater than10^6^ cells/ml, whether exudates of *B. megaterium* may have a dual nature at different concentrations remains to be elucidated. Other factors such as diversity of *B. megaterium* may also resolve the conflicting results between the biocontrol experiments and our study. *B. megaterium* has been reported as a weak plant pathogen^51^,^52^ while in other studies it was shown to be a source of potential plant growth regulators^53^. Genetic differences were found between the isolates used in the *P. capsici* control study^39^ and that used in our study. The former had FAME similarity indices of 52–55% to the type *B. megaterium*^39^, while the latter is 89% similar to the type. Additional investigation with multiple isolates would help to determine whether these differences may result in functional variations.

This study reveals diverse roles of exudates of *B. megaterium* in plant infection by *Phytophthora* species such as triggering quorum sensing-mediated infection and promoting zoospore morphogenesis. The latter as indicated by formation of infection hyphae or appressorium-like structures that are used by fungi and oomycetes to exert pressure to overcome host-cell turgor and/or to secrete enzymes to degrade the plant cell wall and penetrate the cuticle^54^. Appressorium formation in *Phytophthora* species has been related with plant colonization in the early stage of infection^55^. In this study, we found an association between production of such structures and successful plant infection by *P. nicotianae*, suggesting the pathogen colonization promoted plant infection. However, since colonization does not necessarily result in infection, the enhanced infection may be due to a persistent effect of the bacterial exudates, stimulating formation of haustoria that function at the biotrophic stage of infection for effector delivery in the plant cells and pathogen virulence promotion^56^. This is supported by upregulation of RXLR effector genes in the infected host and nonhost plants of *P. sojae* after CFF treatment. Upregulation of these genes often occurs with formation of haustoria during oomycete entry into plant cells to suppress plant defense signaling^57^. In the *P. sojae*-infected host and nonhost plants promoted by CFF, there was upregulation of one or more of these effector genes in inoculated soybean and tobacco plants in which disease development was significantly enhanced in the presence of CFF. L88-8470 was infected with assistance of CFF despite the fact that *Avr3a* but not *Avr1a* corresponding to *RPS1a* was upregulated, and the transcript level of *Avr3a* was not very high. Similarly, CFF treated L83 produced HR with much lower upregulation of *Avr3a,* than *Avr1a* and *Avr3c*, despite correspondence of *Avr3a* with *RPS3a* of the plant. In the infected *N. benthamiana* with CFF, *Avr1a* was upregulated substantially which may give rise to abolishment of the nonhost resistance. Yet, plant resistance genotype is likely a determinant in the assisted interaction between *Phytophthora* and resistant plants by bacteria exudates. For example, in inoculated L92-7857, *RPS3c* may provide an adequate defense to *P. sojae* which was not affected by Sb5; none of the effector genes of the pathogen were upregulated. This suggests that pathogen network mediated modification of effector gene expression contributes to susceptibility of plants which is dependent on genetic background of the plant.

## Materials and Methods

### Soil bacteria isolation, growth conditions, and identities

Bacteria were isolated from soil water extract (SWE). SWE is a solution that is widely used for induction of sporangia and zoospore production due to its containing functional bacterial metabolites and maintaining the activity after autoclaving^8^,^18^,^27^,^28^,^29^. SWE was prepared by mixing 10 g of sandy loam soil from the rhizosphere surface of camellia plants in eastern Virginia with 1 liter of dH_2_O on a magnetic stirrer overnight, followed by filtering through Whatman No. 4 and 1 filter paper. To isolate bacteria, aliquots of 200 μl of SWE were spread on ten 90-mm PDA (potato dextrose agar) plates and incubated at 25 °C for 24 to 48 h. Emerging colonies were grouped by morphology, and representatives of predominant groups were selected, grown on PDA at 25 °C. The Gram nature of these isolates was determined by KOH assay^58^. To prepare fresh cultures, a colony of Gram negative or positive isolates was first grown overnight in a broth of potato dextrose or J (5 g tryptone, 15 g yeast extract, 3 g K_2_HPO_4_, 2 g glucose, pH 7.4)^59^, and then streaked onto the same agar medium plates for 16–24 h at 25 °C. The identities of the isolates were determined by fatty acid methyl ester analysis (FAME) at the Microbial ID laboratory (Newark, DE, USA) and listed in the Table S1.

### Bacterial cell suspension and filtrate preparation

Bacterial cell suspensions were prepared by mixing 10 loops of fresh culture into100 ml sterile distilled water (SDW). This normally gave a cell suspension concentration of 10^7–8^/ml as determined with a Neubauer hemocytometer after a serial dilution. A suspension of 10^7^cells/ml was used as a stock for further applications. Cell suspension filtrates were prepared in slightly different ways depending on experiment. For experiments of leaf disc colonization by *Phytophthora* zoospores, the filtrate was prepared by first agitating the stock suspension overnight, followed by filtering through Whatman 1 and 4 filters and finally autoclaving. For other experiments, cell free filtrate (CFF) was prepared by a centrifugation of the overnight-agitated stock suspension at 5,883 g for 5 min, followed by a filtration of the supernatant through a 0.22-μm filter. For both filtrate preparations, a 1: 10 dilution was made with SDW to get a 10% working concentration.

### Plant species and growth conditions

Plant species used in this study included rhododendron (*Rhododendron catawbiense* “Boursalt”), annual vinca (*Catharanthus roseus* cv. Little Bright Eye), tobacco (*Nicotiana benthamiana*), *Arabidopsis thaliana* Col-0, and soybean (*Glycine max*) differential plants for *P. sojae* race 25: Williams, L88-8470 (*RPS1a*), L77-1863 (*RPS1b*), L83-570 (*RPS3a*) and L92-7857 (*RPS3c*). Williams and L77-1863 are susceptible to the race while the other differentials are resistant^60^.

Annual vinca was seeded into Metro Mix 360 and grown on a mist bench for 5 weeks, then transplanted into pine bark and grown for an additional 4 to 6 weeks in a greenhouse. Plants after transplanting were fertilized twice with 250 mg/L liquid 20-20-20 (20% N- 20% P_2_O_5_-20% K_2_O; Scotts, Marysville, OH). *Arabidopsis,* tobacco, and soybean were seeded in Metro-Mix 200 or Metro-Mix 360 (Scotts, Marysville, OH). Soybean plants were grown in a growth chamber at 25 °C with a 14/10 h light/dark cycle for 7 to 10 days before use. Tobacco and *Arabidopsis* plants were grown for 4 to 5 weeks under the same condition as for the soybean and fertilized once with the liquid 20-20-20 one week before use. Rhododendron plants were obtained from Briggs Nursery in Washington, USA and their leaf discs were prepared as described previously^61^.

### *Phytophthora* species, growth conditions and zoospore suspension preparation

Thirteen *Phytophthora* species/isolates were used in this study. Apart from those listed in Table 1, *P. sojae* isolate 28G4 (P7081, race 25) and *P. nicotianae* 1B11 were used extensively. All isolates were cultured in 10% clarified V8 broth or on 5% V8 agar at 23–25 °C. Zoospore suspensions were prepared from cultures in broth medium or 2 to 6 day-old agar culture plugs with methods described previously^18^. A zoospore suspension of 10,000/ml SDW was used as a stock for further dilution into the various solutions including autoclaved SWE (ASWE), filtrates of suspensions or CFF of selected bacterial isolates, fractionated CFF, SDW as well as rinsate of agar medium surface with 10 ml of SDW (SDWms).

### Colonization of rhododendron leaf discs by multiple *Phytophthora* species

Leaf discs of rhododendron, a plant susceptible to numerous *Phytophthora* species^8^, were used to investigate the effects of five selected dominant soil bacterial isolates on *Phytophthora* zoospore colonization. Leaves were surface sterilized with 75% ethanol to limit effects of other microorganisms and cut into 4-mm diameter discs. 15–20 of the discs were submerged in 100-ml plastic bottles with a 60 ml zoospore suspension at 10/ml in a bacterial suspension filtrate, ASWE, SDW or SDWms. The discs were kept in the solutions at 23 °C overnight then plated on PARP-V8 agar, a *Phytophthora* selective medium^62^ for an additional 48 h before determination of pathogen colonization. Leaf discs colonized by *Phytophthora* were counted. Each experiment included three replicates and was repeated at least once.

### Phytopathosystems and infection assays with *P. nicotianae* and *P. sojae*

Three phytopathosytems, annual vinca - *P. nicotianae*, soybean - *P. sojae,* and tobacco - *P. sojae* were used. In the annual vinca - *P. nicotianae* system, a single zoospore infection assay was conducted with detached leaves as described in a previous study^11^. Briefly, zoospores were suspended in CFF, SDW or ASWE to be tested to obtain an inoculum concentration of 100/ml. To make single zoospore inoculation, 10-μl drops of the inoculum were placed on 10 different locations on the surface of a leaf and incubated at 23 °C. Infected sites were counted 72 h post inoculation. The experiment included 18 leaves or 180 inoculated sites and was repeated twice.

In the soybean - *P. sojae* system, the hypocotyl inoculation method was performed as described previously^60^. Specifically, *P. sojae* 28G4 inocula were prepared with a 2-day-old mycelial mat grown in V8 broth which was washed twice with SDW and drained to remove the nutrients from the media. At inoculation, 50 mg of mycelia was mixed with 1 ml of CFF or SDW and macerated with a syringe. Each hypocotyl was injected with 20 μl of the slurry and kept in a moist chamber at 23–25 °C for 2–4 days. Inoculated plants were measured for infection based on presence of a dark lesion and lesion sizes at the inoculation site after 3 days. Nine replicates were included and assay was repeated twice.

In the tobacco - *P. sojae* system, both detached leaves and plant seedlings were used. To inoculate detached leaves, a 20-μl drop of a zoospore suspension at 12,500/ml SDW or CFF was placed on 4 sites of the abaxial side of a leaf. Inoculated leaves were rated for disease incidence based on occurrence of yellowing or blight symptoms after 72 h at 25 °C. Each assay included 15 replicate leaves and was repeated once. To inoculate seedlings, roots first were rinsed off Metro Mix with tap water and SDW, respectively, and were then placed in a 30-ml beaker with10 ml zoospore suspension at 3,200/ml SDW, a live Sb5 cell suspension at 10^6^/ml or a mixed suspension of zoospore and bacteria at the same individual concentrations. To determine a concentration effect of bacterial cells, a 10-fold dilution series of Sb5 from 10^6^/ml was used. Plants were kept in the dark overnight after inoculation then incubated for an additional 6 days under the plant growth conditions before disease assessment. Plants showing dieback or blight symptoms were recorded as diseased. Each assay included 3–7 plant replicates depending on experiment and was repeated at least once.

To determine the causal agent of the inoculated tobacco with Sb5 and/or *P. sojae*, symptomatic plant tissues were surface sterilized with 10% bleach to eliminate attached microorganisms on the surface and prevent false positive. They were then plated on PARP-V8 and J- agar at 23 or 25 °C respectively, in the dark for 3 days to allow growth of the causal agent from inside of plant tissues. Mycelial or bacterial growth from the tissue was recorded. Growing mycelia were used to prepare DNA to determine the identity with ITS1-SSCP analysis for *Phytophthora* species^20^.

### Microscopy of effects of Sb5 exudates on zoospore behaviors

Zoospore homing behaviors including motility, plant tissue attachment and cyst germination in response to CFF were examined under an Olympus IX71 inverted microscope at magnification of 40–160×. Experiments investigating zoospore motility and cyst germination were conducted *in vitro*. Approximately 500 zoospores were suspended in 100 μl CFF or SDW in a depression slide and incubated at 23 °C for 2, 4, 6, and 16 h. To assess mobility of zoospores, quantity of swimming zoospores in each of three microscopic fields was estimated at each time point and compared to that at zero point and duration between treated and untreated zoospores. Similarly, to determine germination in response to CFF, germinated cysts in both treatments were rated in three fields 16 h after incubation. Each observation included two replication slides.

Experiments for zoospore attachment were conducted with detached clean roots of 5-week old *Arabidopsis* plants. Individual roots were submerged in a 50 ml zoospore suspension of *P. nicotianae* or *P. sojae* at 10,000/ml SDW or CFF for 40 min at 23 °C and then rinsed with water to remove unattached zoospores. Plant tissue was examined for cyst attachment after stained with trypan blue solution^63^. Two randomly selected roots were observed. Attached cysts were counted in three fields of the microscope.

### Analyses of gene expression of RXLR effectors in response to Sb5 exudates

Experiments of expression of RXLR effector genes of *P. sojae* in the inoculated plants were conducted under the MIQE guidelines^64^. Samples were taken from soybean 2-cm sections of inoculated sites from five hypocotyls or three roots of inoculated tobacco seedlings, ground in liquid nitrogen and stored at −80 °C after plants were inoculated for 48 h with *P. sojae* in the presence or absence of CFF or Sb5 as described above. RNA was extracted from the frozen samples with the RNeasy Mini Kit and RNase-Free DNase Set (Qiagen, Valencia, CA). cDNA was obtained by reverse transcription of 5 μg of RNA with the High Capacity cDNA Archive Kit (Life Technologies Carlsbad, CA). 2.5 μl of the cDNA of each sample or the PCR negative control dH_2_O was amplified in a 25 μl PCR reaction with a pair of primers listed in Table S10 and the Power SYBR^®^ Green PCR Master Mix in the ABI 7500 Real Time PCR System (Life Technologies Carlsbad, CA). Each sample was run for at least three times at the 9700 mode with 50 °C for 2 min at first stage, 95 °C for 10 min at second stage followed by 40 cycles of 95 °C for15 sec and 60 °C for 1 min.

Gene expression was based on Ct values (threshold cycle), standard errors of Ct (Ct std err) and average Ct (AvgCt) of genes in each sample from the PCRs as determined with the Relative Quantification Study of the software SDS v.1.3.1 at the default setting (Life Technologies Carlsbad, CA) (Table S7). To get relative expression levels of a target gene, AvgCt of the target gene in a Sb5 treated or untreated sample was normalized against AvgCt of the mean of AvgCt of the three reference genes (*Actin A*, *β-tubulin* and *ubiquitin*) (Table S10) in the same sample, or dAvgCt  (=AvgCt_target_ − AvgCt_refs_). To obtain expression levels of the target genes in treated samples, dAvgCt of treated samples were calibrated with dAvgCt of untreated to get ddAvgCt  (=AvgCt_Sb5_ − AvgCt_SDW_) followed by quantification with 2^−ddAvgCT^. The maximum and minimum ddAvgCt was calculated based on the standard errors of dCt (Table S7).

### Identification of Sb5 and fractionation of CFF

In addition to (FAME), the identity of Sb5 was determined by sequencing the amplicons of 16S rDNA of the isolate with primers 968F and 1410R^65^. Both strands of the sequences were processed with BioEdit V7.2.5 (http://www.mbio.ncsu.edu/BioEdit/bioedit.html)^66^ and BLASTed against 16S rDNA sequences of microbes at http://blast.ncbi.nlm.nih.gov to match to a species level. The final sequence was then deposited into GenBank.

Sb5 CFF was fractionated with centrifugal filter columns to estimate the sizes of active components. CFF was first loaded into Centriprep YM-3 (MWCO 3 kDa) and centrifuged as instructed by the manufacture (Millipore.com). The filtrate was designated as <3 kDa fraction. The remaining CFF in the column was subsequently loaded to YM-10 (MWCO 10 kDa) and centrifuged. The filtrate collected was designated as 3–10 kDa fraction while the remaining in the second column was processed with the third column, Centriprep YM-30. The resulting filtrate was designated as 10–30 kDa fraction and the solution that did not go through was designated as >30 kDa fraction. These four fractions were then tested together with the controls CFF and SDW for bioactivities in the microscopy and infection assays described above.

### Statistical analyses

Proc ANOVA in SAS with least significance difference (LSD) and t-Test at equal and unequal variances in Excel were used for evaluating statistical significance and comparison of means between treatments. *P*-value < 0.05 was considered significant. Standard errors were calculated based on the standard deviation of the replicates.

## Additional Information

**How to cite this article**: Kong, P. and Hong, C. Soil bacteria as sources of virulence signal providers promoting plant infection by *Phytophthora* pathogens. *Sci. Rep.* **6**, 33239; doi: 10.1038/srep33239 (2016).

## Supplementary Material

Supplementary Information

Supplementary Table S1

Supplementary Table S2

Supplementary Table S3

Supplementary Table S4

Supplementary Table S5

Supplementary Table S6

Supplementary Table S7

Supplementary Table S8

Supplementary Table S9

## Figures and Tables

**Table 1:** Effect of Sb5 cell suspension filtrate on colonization of rhododendron leaf discs (%) by zoospores of *Phytophthora* species.

Species	Isolate^x^	Source	Provider^y^	Colonization (%) of leaf discs in following solutions^z^			*P* value
				Sb5	ASWE	SDW	
*P. cactorum*	29G7	*Rhododendron* sp.	CH	84.4 a	91.1 a	0.0 b	0.0008
*P. capsici*	22H3 (p62)	*Cucurbita* sp.	MG	97.8 a	100.0 a	8.9 b	<0.0001
*P. cinnamomi*	30D6	*Ilex glabra*	CH	88.9 a	77.8 a	6.7 b	0.0016
*P. cryptogea*	15E6 (D200)	Soil	SJ	93.3 a	68.9 a	6.7 c	<0.0001
*P. hydropathica*	1D12	Irrigation water	CH	97.8 a	100.0 a	17.8 b	<0.0001
*P. megasperma*	23A3 (MYA-3660)	*Actinidia chinensis*	MG	24.4 ab	53.3 a	2.2 b	0.0232
*P. nicotianae*	IE2 (GLN10-5)	Irrigation water	SB	71.7 a	93.3 a	10.0 b	0.0011
*P. palmivora*	7A9	Irrigation water	CH	100.0 a	100.0 a	0.0 b	<0.0001
*P. pini*	22F1 (MYA3656)	*Rhododendron* sp.	MG	100.0 a	100.0 a	0.0 b	<0.0001
*P. sojae*	28G3 (p9073)	*Glycine max*	BT	37.8 a	35.6 a	0.0 b	0.0176
*P. tropicalis*	22H5 (p27)	*Vanilla* sp.	MG	100.0 a	100.0 a	13.3 b	<0.0001

^x^The original isolate code from the provider is listed in the parenthesis.

^y^BT = Brett Tyler at Oregon State University, CH = Chuanxue Hong at Virginia Tech, MB = Michael Benson at North Carolina State University, MG = Mannon Gallegly at West Virginia University, SJ = Steve Jeffers at Clemson University, and SB = Sharon Von Broembsen at Oklahoma State University.

^z^Average colonization (%) of three replicates; each included 15~20 rhododendron leaf discs in autoclaved filtrate of Sb5 cell suspension diluted 1:10, autoclaved soil water extract (ASWE) or sterile distilled water (SDW) containing 10 zoospores/ml. The numbers in rows followed by the same letter did not differ according to LSD test at *P* = 0.05.
