# Supplementary material for: Soil bacteria as sources of virulence signal providers promoting plant infection by Phytophthora pathogens
Source: Sci Rep. 2016 Sep 12;6:33239. doi: 10.1038/srep33239 (PMC5018965; doi:10.1038/srep33239)

Title: Soil bacteria as sources of virulence signal providers promoting plant infection by *Phytophthora* pathogens

Authors: Ping Kong and Chuanxue Hong

Supporting information

Table S1-9. Correspondence of Table S containing dataset/ statistics in separate Excel files to Figs. 1-7 and Table 1

Table S	1	2	3	4	5	6	7	8	9
Fig.	1	2b	3b	4a	4c	5b	6b	7	Table1

Table S10 Oligonucleotide primers used in this study

Gene origin	Primer name	Sequence (5' - 3')	Reference
P. sojae			
Avr1a	Avr1a R2	GTCTTTCAAATCGTCGCTCAATT	Qutob *et al*, 2009
	ATG Avr1a-F	CAGTTATCAAGAGCCCGACCA	
Avr3a	Sp92-33F	GCTGCTTCCTTCCTGGTTGC	Qutob *et al*, 2009
	Sp92-325R	GCTGCTGCCTTTTGCTTCTC	
Avr1b-1	Avr1bReF	ACCTTCAGCGTGACTGACCT	Dou *et al*, 2008
	Avr1bReR	GCGATTGCCAACCAGTTCT	
Avr3c	Avh27aRT-F	TCAAAAAGTGGATAGAAGAAAAAC	Dong *et al*, 2009
	Avh27aRT-R	ACCCACGCTTTGTTTAGTCTCT	
ActinA	ActinF	CGACATCCGTAAGGACCTGT	Dong *et al*, 2009
	ActinR	TTCGAGATCCACATCTGCTG	
Ubiquitin	UBF	GCAACTCGCTTCCACGA	Tyler’s laboratory,2004
	UBR	GAACCAGCAACACTTGAT	
Phytophthora			
β-tubulin	TUB1	GATCCCGTTCCCGCGTCT	Ma, *et al*, 2009
	TUB2	CGCTTGAACATCTCCTGGAT	

Fig. S1 DNA fingerprinting of *Phytophthora sojae* isolates from diseased *Nicotiana benthamiana* plants. *N. benthamiana* roots were inoculated with *P. sojae* zoospore suspension at 3,200/ml containing Sb5 cells at 10^6 /ml. Eleven *Phytophthora* isolates were recovered from diseased tissue after surface-sterilization. These isolates (lanes 1-11) were identified with PCR-SSCP (single strand DNA conformational polymorphisms) of internal transcribed spacer one (ITS1). CK+ is a positive control from *P. sojae* and CK- is a negative control from SDW. M is a SSCP size marker.

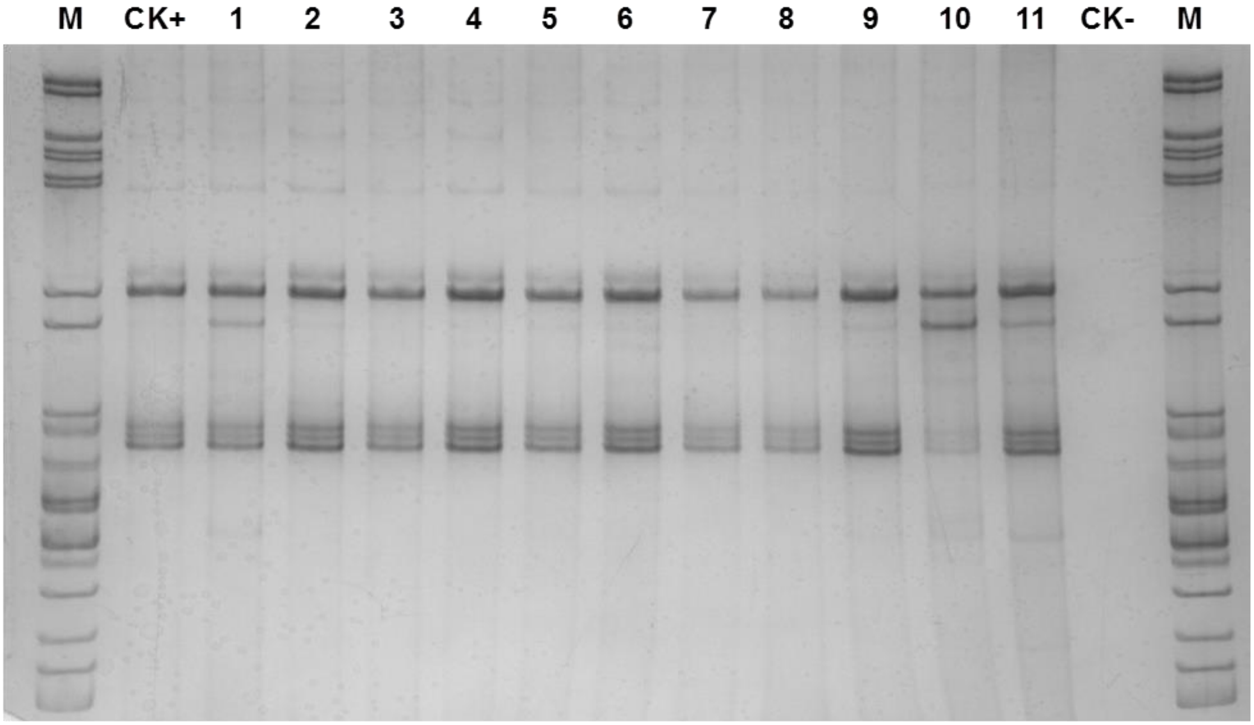

Fig. **S2.** Photomicrographs of morphogenesis of *Phytophthora* zoospores in response to Sb5 cell-free filtrate (CFF). Zoospores germinated and formed finger-like projections or vesicles in CFF but not in SDW after a14-h incubation at 23ºC. *c, p* and *v* represent cyst, finger-like projections or vesicles, respectively. Bars = 50 µm.

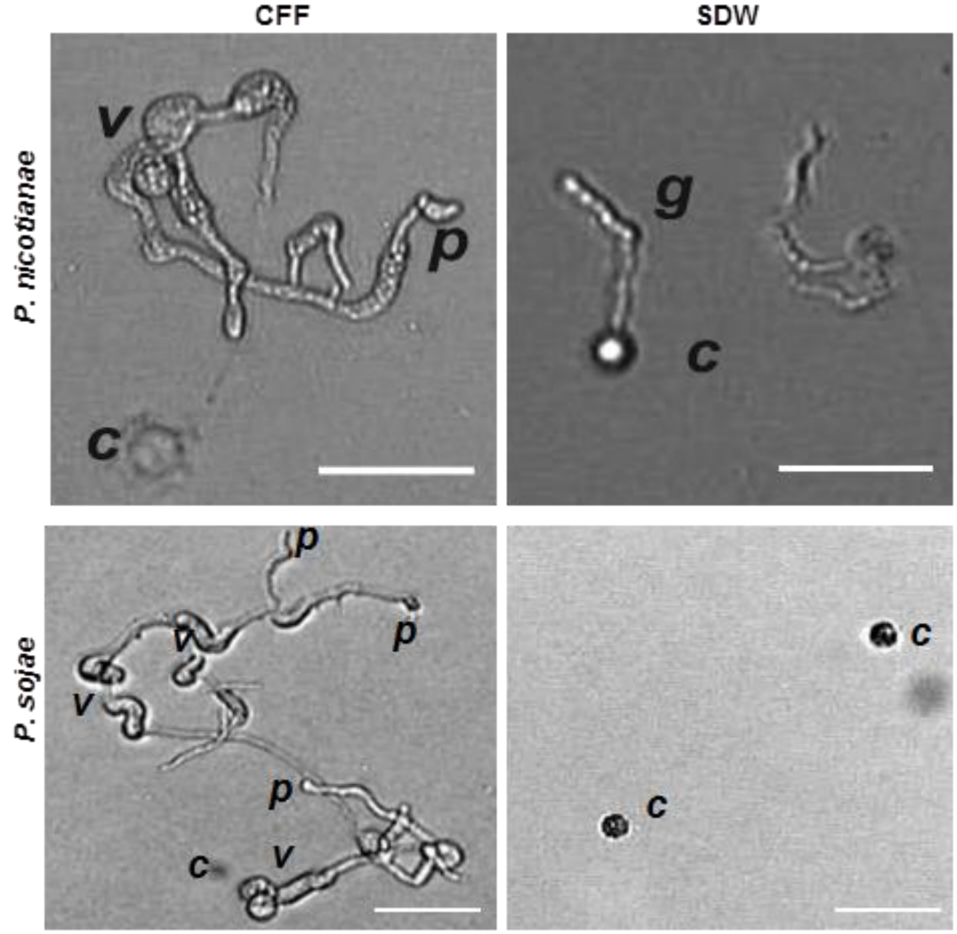

Supplement: Supplementary Information [file srep33239-s1.pdf]
